# Association of Life’s essential 8 score with the risk of all-cause mortality and cardio-cerebrovascular mortality in patients with stroke

**DOI:** 10.1186/s12872-024-03947-3

**Published:** 2024-06-25

**Authors:** Bin Yan, Yan Jin, Song Mao, Yugang Yin

**Affiliations:** 1 Department of Geriatric Neurology, Jinling Hospital, Medical School of Nanjing University, Nanjing, 210002 Jiangsu Province People’s Republic of China; 2 Department of Pharmacy, Jinling Hospital, Medical School of Nanjing University, Nanjing, 210002 Jiangsu Province People’s Republic of China; 3 Department of Pediatrics, Jinling Hospital, Medical School of Nanjing University, Nanjing, 210002 Jiangsu Province People’s Republic of China; 4 Department of Geriatric Cardiology, Jinling Hospital, Medical School of Nanjing University, No.305 Zhongshan East Road, Xuanwu District, Nanjing, 210002 Jiangsu Province People’s Republic of China

**Keywords:** Life's Essential 8, Stroke, All-cause mortality, Cardio-cerebrovascular mortality, NHANES

## Abstract

**Background:**

A higher Life's Essential 8 (LE8)-based cardiovascular health (CVH) has been reported to be associated with a lower risk of both all-cause mortality and cardio-cerebrovascular diseases (CCVDs) related mortality in adults in the United States. At the same time, multiple studies have shown a significant negative association of CVH with the risk of stroke and CCVDs. Since no research has investigated the applicability of the LE8 in stroke patients, this study aimed to explore the association of LE8 with all-cause mortality and cardio-cerebrovascular mortality in stroke patients.

**Methods:**

Data of patients were extracted from the National Health and Nutrition Examination Surveys (NHANES) database in 2007–2018 in this retrospective cohort study. Weighted univariate and multivariate COX regression analyses were utilized to investigate the associations of LE8 with all-cause mortality and cardio-cerebrovascular mortality. We further explored these relationships in subgroups of age, gender, body mass index (BMI), cancer, congestive heart failure (CHF), and coronary heart disease (CHD). The evaluation indexes were hazard ratios (HRs) and 95% confidence intervals (CIs).

**Results:**

Among the eligible patients, 278 died from all-cause and 89 (8.38%) of them died due to CCVDs. After adjusting for covariates, patients with LE8 score ≥ 58.75 seemed to have both lower risk of all-cause mortality (HR = 0.46, 95%CI: 0.31–0.69) and cardio-cerebrovascular mortality (HR = 0.51, 95%CI: 0.26–0.98), comparing to those with LE8 score < 48.123. Higher LE8 scores were associated with lower risk of all-cause mortality in patients aged < 65 years old, without cancer, and whatever the gender, BMI, CHF or CHD conditions (all *P* < 0.05). The relationships between high LE8 scores and low cardio-cerebrovascular mortality risk were only found in age < 65 years old and non-cancer subgroups (all *P* < 0.05).

**Conclusion:**

A higher LE8 score was associated with lower risk of both all-cause mortality and cardio-cerebrovascular mortality in patients with stroke, which may provide some reference for risk management and prognosis improvement in stoke. However, more evidences are needed to verify this beneficial role of high LE8 score in stroke prognosis.

**Supplementary Information:**

The online version contains supplementary material available at 10.1186/s12872-024-03947-3.

## Background

Stroke is one of the most well-known and extensively studied cardio-cerebrovascular diseases (CCVDs), which is the second cause of mortality and the third cause of disability worldwide [1, 2]. In 2019, there were 12.2 million new stroke cases and the dead cases up to 6.55 million, contributing to a significant global disease burden [3]. Therefore, prevention and management of stroke has become a major public health challenge in the world today.

In recent years, influencing factors associated with prognoses in patients with stroke have been explored widely. The main contributors to CCVDs include smoking, alcohol intake, hypertension, diabetes mellitus (DM), dyslipidaemia and atrial fibrillation, and the most prevalent risk factors among stroke survivors were hypertension and smoking [4]. Also, physical activity as well as diet quality could significantly modified the negative impact of comorbidity on disability in stroke patients [5]. In 2010, a composite indicator basing on seven healthy behaviors and health factors was constructed by the American Heart Association (AHA), namely the Life's Simple 7 (LS7), to represent cardiovascular health (CVH) conditions [6]. The LS7 aims to facilitate a shift from focusing solely on the treatment of disease to actively promoting and maintaining health throughout the life course of populations and individuals [6]. The AHA updated this definition according to experience and evidences in 2022, adding sleep to construct the Life's Essential 8 (LE8), which includes diet quality, physical activity, tobacco exposure, sleep health, body mass index (BMI), lipids, blood sugar and blood pressure [7]. Population-based evidences have suggested a higher LE8-based CVH is linked to lower risk of both all-cause mortality and CCVD-related mortality in the United States adults [8–10]. Besides, multiple prospective cohort studies have shown a significant negative association of LE8 with the risk of stroke and cardiovascular diseases [11, 12]. However, since no research has investigated the applicability of the LE8 in outcomes of stroke patients, it is necessary to clarify whether LE8 could be beneficial for prognoses management in stroke survivors, or bring about extraneous earnings.

Herein, the current study aims to explore the associations of the LE8 with all-cause mortality and cardio-cerebrovascular mortality in patients with stroke, with the hope of providing some references for health management and the reduction of disease burden in stroke.

## Methods

### Study design and participants

This was a retrospective cohort study, and data of patients with stroke were extracted from the National Health and Nutrition Examination Surveys (NHANES) database in 2007–2018. The NHANES is conducted jointly by the National Center for Health Statistics (NCHS) and the Centers for Disease Control and Prevention (CDC) that aims to assess nutritional and health status of noninstitutionalized population in the United States. It includes a complex, multistage stratified probability sample on the basis of selected counties, blocks, households, and persons within households. Information was collected through interviews in participants’ homes that conducted by the NCHS well trained professionals, and extensive physical examinations conducted at mobile exam centers (MECs). For details please visit: https://www.cdc.gov/nchs/nhanes/index.htm.

There were 1,398 individuals diagnosed as stroke in the database. The exclusion criteria were (1) aged < 20 years old, (2) missing information on LE8 assessment, (3) missing survival data, and (4) missing information on study variables including education level, sedentary time, cancer, congestive heart failure (CHF), coronary heart disease (CHD), time course of stroke and cotinine. Finally, 865 patients were eligible. The NHANES survey has been approved by the institutional review board (IRB) of the NCHS, and all the participants have provided informed consent. The requirement of ethical approval for this study was waived by the IRB of Jinling Hospital, Medical School of Nanjing University, because this database was publicly available. In addition, all study methods were performed in accordance with the relevant guidelines and regulations.

### Measurement of LE8

The LE8 score is composed of four health behaviors (including diet quality, physical activity, tobacco exposure and sleep duration) as well as four health factors (BMI, non-high-density-lipoprotein cholesterol [non-HDL-C], blood glucose and blood pressure). Detailed algorithms for calculation of the LE8 scores for each of the metrics to NHANES data have been shown elsewhere [13]. Briefly, each of the eight factors was scored ranging from 0 to 100 points, and the unweighted average of these factors were used to calculate the overall LE8 score. Therefore, a higher LE8 score represents a better health condition. In the current study, we categorized the LE8 score (< 48.13, 48.13–58.75 and ≥ 58.75), health behaviors (< 43.75, 43.75–61.25 and ≥ 61.25), and health factors (< 47.50, 47.50–62.50 and ≥ 62.50) into three levels according to their tertiles respectively [7].

In addition, we calculated the contribution of each component in LE8 to reflect importance rank of these variables. To be specific, random forest models were established to analyses associations of each component in LE8 with all-cause mortality and cardio-cerebrovascular mortality respectively, and using variable importance (VIMP) method to rank the importance of these components. The VIMP calculated the difference value of error rate in models before and after inclusion of the variables. If a variable had a VIMP value < 0 represents it reduced the accuracy prediction of the model. Also, the larger the VIMP value, the greater the impact of this variable on the accuracy of the model, and the greater the importance of this variable.

### Variables selection

We also extracted variables from the database as potential covariates, including age, gender, race, education level, marital status, poverty income ratio (PIR), heavy alcohol drinking, sedentary time, hypertension, dyslipidemia, DM, depression, cancer, CHF, CHD, anticoagulants, antiplatelet agents, sleep duration, time course of stroke, height, weight, BMI, waist circumference, systolic blood pressure (SBP), diastolic blood pressure (DBP), total cholesterol (TC), low-density-lipoprotein cholesterol (LDL-C), HDL-C, non-HDL-C, triglyceride (TG), fasting glucose, glycosylated hemoglobin (HbAlc), creatinine (Cr), estimated glomerular filtration rate (eGFR) and the Healthy Eating Index (HEI)-2015.

In the LE8 scoring algorithm, diet quality was evaluated using the HEI-2015. The NHANES collected dietary intakes of participants via two 24-h dietary recalls, and was combined with the United States Department of Agriculture (USDA) food patterns equivalents data to construct and calculate the HEI-2015 scores [14]. Information on heavy alcohol drinking (≥ 8 drinks/week) was collected by self-report questionnaires [15]. DM, dyslipidemia, and hypertension were estimated through laboratory examination, self-report, or medication history: participants with fasting blood glucose ≥ 7.0 mmol/L or HbAlc ≥ 6.5% or self-reported DM or receiving hypoglycemic therapy were considered as DM patients; dyslipidemia referred to TC ≥ 200 mg/dL (5.2 mmol/L) or TG ≥ 150 mg/dL (1.7 mmol/L) or LDL-C ≥ 130 mg/dL (3.4 mmol/L) or HDL-C ≤ 40 mg/dL (1.0 mmol/L) or self-reported hypercholesterolemia or lipid-lowering therapy; hypertension was defined as self-reported high blood pressure or SBP ≥ 130 mmHg or DBP ≥ 80 mmHg or taking hypotensive drugs [16]. In addition, cancer, stroke, depression were also assessed via the NHANES questionnaires [17].

Height, and weights were measured during the physical examination in MECs. The BMI was calculated as the weight in kilograms (kg) divided by the height in meters squared (m^2^). According to the World Health Organization (WHO) standard, we divided BMI into underweight (< 18.5 kg/m^2^), normal weight (18.5 kg/m^2^ ≤ BMI < 25 kg/m^2^), overweight (25 kg/m^2^ ≤ BMI < 30 kg/m^2^), and obesity (BMI ≥ 30 kg/m^2^). Besides, the cut-off value for high waist circumference was 88 cm for females and 102 cm for males [18]. Also, the blood samples were collected and sent to central laboratories for the determination of hematological indexes in the NHANES.

### Study outcomes and follow-up duration

The study outcomes were all-cause mortality and cardio-cerebrovascular mortality. The NHANES public-use linked mortality file as of December 31, 2019, which was correlated with the NCHS with the National Death Index (NDI) through a probability matching algorithm was used to determine the mortality status of the participants (https://ftp.cdc.gov/pub/health_statistics/NCHS/datalinkage/linked_mortality/). Of which, dying from arbitrary cause was recognized as all-cause mortality, whereas dying due to “disease of heart” (I00-I09, I11, I13, I20-I51) or “cerebrovascular diseases” (I60-I69) was considered as cardio-cerebrovascular mortality. Moreover, the follow-up ended when participants died or at December 31, 2019.

### Statistical analysis

Normally distributed continuous data were described using mean ± standard error (mean ± SE), and t test was used for comparation between survival group and all-cause mortality group. Categorical data were expressed as frequency and constituent ratio [N (%)], and chi-square test (χ^2^) was used for comparison. Due to we included information collected from two 24-h dietary recalls, special weights “dietary two-day sample weight (WTDR2D)” should be used according to the NHANES guideline. The WTDR2D weights were constructed by taking the MEC two-year cycle sample weights (WTMEC2YR), and further adjusting for (a) the additional non-response and (b) the differential allocation by day of the week for the dietary intake data collection.

Weighted univariate COX regression analyses were used to screen the covariates associated with all-cause mortality and cardio-cerebrovascular mortality respectively. Weighted univariate and multivariate COX regression analyses were employed to investigate the association of LE8 with all-cause mortality and cardio-cerebrovascular mortality in patients with stroke. Also, these relationships were assessed in subgroups of age, gender, BMI, cancer, and CHD. Model 1 was unadjusted model. Model 2 adjusted for demographic and socioeconomic factors, including age, gender, race, educational level, marital status and PIR. Model 3 additionally adjusted for selected covariates on the basis of Model 2. The evaluation indexes were hazard ratios (HRs) and 95% confidence intervals (CIs). Two-sided* P* < 0.05 was considered significant. Statistical analyses were performed by SAS 9.4 (SAS Institute, Cary, NC, USA) and R version 4.2.3 (Institute for Statistics and Mathematics, Vienna, Austria).

## Results

### Characteristics of participants

**Fig. 1:**
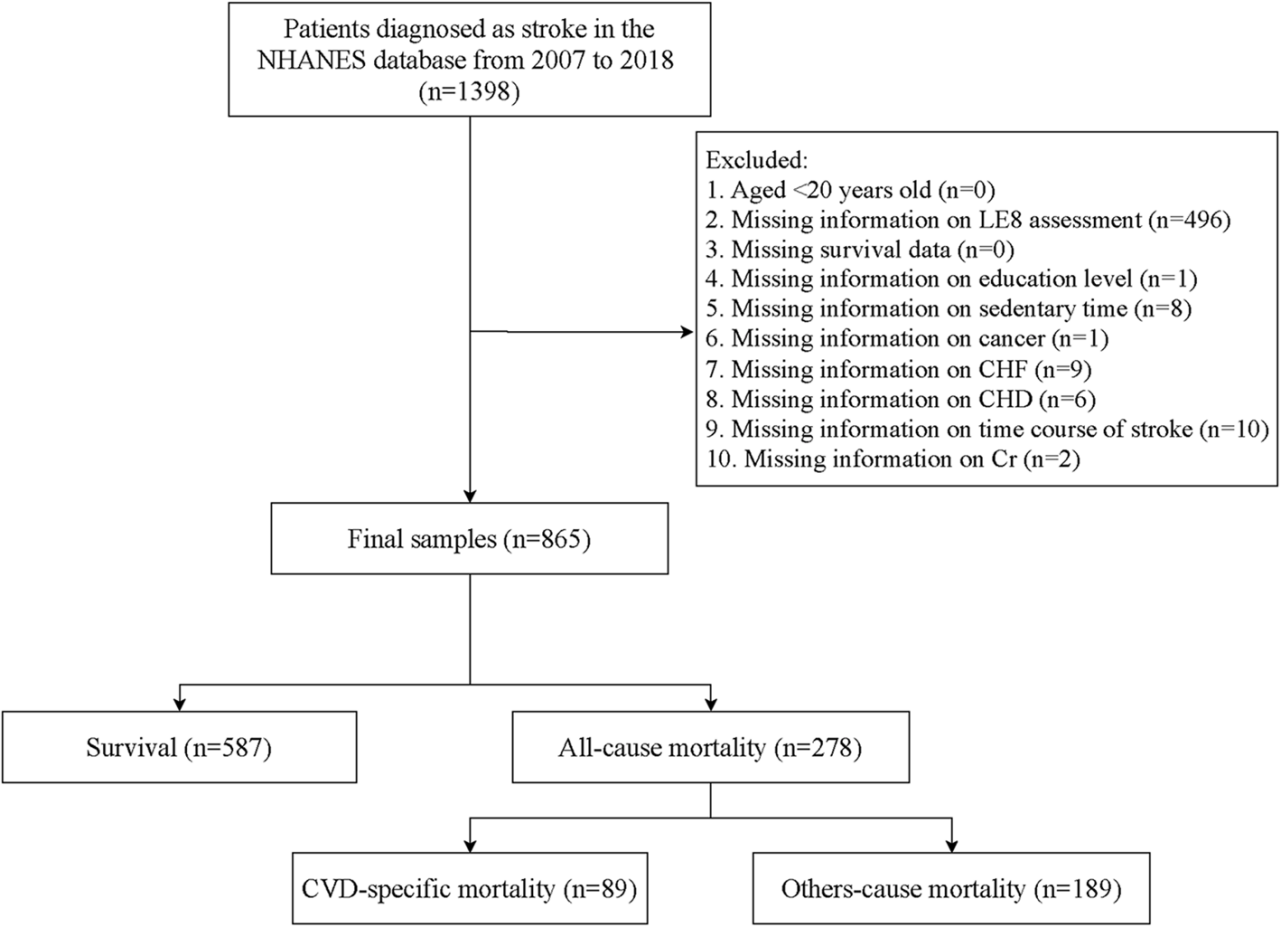
Flowchart of stroke patients screening

The procedure of study participants screening was shown in the Fig. 1. We initially included 1,398 patients with stroke in the NHANES. Then, those who missing information on LE8 assessment (*n* = 496), education level (*n* = 1), sedentary time (*n* = 8), cancer (*n* = 1), CHF (*n* = 9), CHD (*n* = 6), time course of stroke (*n* = 10) or Cr (*n* = 2) were excluded. Finally, 865 were eligible.

The median follow-up duration was 64 months, and a total of 278 patients died from all cause. The comparation on characteristics of patients between survival group and all-cause mortality group was shown in the Table 1. Average age of the study population was 63.66 years old, and 451 (57.45%) were female. In survival group, most patients had LE8 score ≥ 58.75 [210 (42.68%)], followed by that < 48.13 [206 (31.13%)], whereas in all-cause mortality group, most patients had LE8 score < 48.13 [95 (33.81%)], and followed by that ≥ 58.75 [91 (33.44%)]. However, between survival group and all-cause mortality group, no significant difference was found in LE8 score, health behaviors score or health factors score (all *P* > 0.05). In addition, list of the score of each component in LE8 was shown in the Table S1.

**Table 1:** Characteristics of eligible patients with stroke between survival group and all-cause mortality group

Variables	Total (*N* = 865)	Survival (*N* = 587)	All-cause mortality (*N* = 278)	Statistics	*P*
Age, years, Mean ± SE	63.66 ± 0.87	60.42 ± 1.07	71.82 ± 1.06	t = 7.002	< 0.001
Gender, n (%)				χ^2^ = 0.377	0.541
Female	451 (57.45)	325 (58.36)	126 (55.15)		
Male	414 (42.55)	262 (41.64)	152 (44.85)		
Race, n (%)				χ^2^ = 5.350	0.002
Non-Hispanic White	449 (70.73)	264 (66.73)	185 (80.83)		
Non-Hispanic Black	236 (15.39)	178 (16.63)	58 (12.25)		
Mexican American	75 (5.22)	57 (5.98)	18 (3.33)		
Others	105 (8.66)	88 (10.66)	17 (3.59)		
Educational level, n (%)				χ^2^ = 3.628	0.031
Less than high school	288 (27.26)	182 (24.13)	106 (35.15)		
High School	234 (29.07)	155 (29.40)	79 (28.22)		
Above high school	343 (43.68)	250 (46.47)	93 (36.63)		
Marital status, n (%)				χ^2^ = 3.512	0.064
Married/Living with partner	471 (61.10)	330 (64.09)	141 (53.57)		
Never married/divorced/separated/widowed	394 (38.90)	257 (35.91)	137 (46.43)		
PIR, n (%)				χ^2^ = 2.198	0.118
≤ 1.3	315 (29.47)	229 (31.31)	86 (24.81)		
> 1.3	496 (63.82)	325 (63.43)	171 (64.79)		
Unknown	54 (6.72)	33 (5.25)	21 (10.40)		
Heavy alcohol drinking, n (%)				χ^2^ = 0.150	0.853
No	535 (61.16)	367 (60.83)	168 (62.01)		
Yes	53 (8.94)	36 (9.41)	17 (7.74)		
Unknown	277 (29.90)	184 (29.76)	93 (30.25)		
Sedentary time, hours/day, n (%)				χ^2^ = 0.591	0.444
< 4	172 (16.24)	128 (17.06)	44 (14.19)		
≥ 4	693 (83.76)	459 (82.94)	234 (85.81)		
Hypertension, n (%)				χ^2^ = 10.132	0.002
No	76 (12.97)	65 (16.35)	11 (4.43)		
Yes	789 (87.03)	522 (83.65)	267 (95.57)		
Dyslipidemia, n (%)				χ^2^ = 0.797	0.374
No	84 (10.23)	54 (9.51)	30 (12.03)		
Yes	781 (89.77)	533 (90.49)	248 (87.97)		
DM, n (%)				χ^2^ = 1.373	0.245
No	520 (65.28)	365 (66.69)	155 (61.71)		
Yes	345 (34.72)	222 (33.31)	123 (38.29)		
Depression, n (%)				χ^2^ = 4.408	0.039
No	562 (64.83)	368 (62.16)	194 (71.57)		
Yes	303 (35.17)	219 (37.84)	84 (28.43)		
Cancer, n (%)				χ^2^ = 26.337	< 0.001
No	672 (78.41)	484 (84.29)	188 (63.57)		
Yes	193 (21.59)	103 (15.71)	90 (36.43)		
CHF, n (%)				χ^2^ = 5.805	0.018
No	713 (82.85)	503 (85.54)	210 (76.04)		
Yes	152 (17.15)	84 (14.46)	68 (23.96)		
CHD, n (%)				χ^2^ = 7.088	0.009
No	705 (79.71)	495 (83.20)	210 (70.90)		
Yes	160 (20.29)	92 (16.80)	68 (29.10)		
Anticoagulants, n (%)				χ^2^ = 9.619	0.003
No	748 (88.82)	530 (91.65)	218 (81.68)		
Yes	117 (11.18)	57 (8.35)	60 (18.32)		
Antiplatelet agents, n (%)				χ^2^ = 3.829	0.054
No	663 (76.05)	462 (78.40)	201 (70.12)		
Yes	202 (23.95)	125 (21.60)	77 (29.88)		
Sleep duration, hours, Mean ± SE	7.30 ± 0.10	7.32 ± 0.12	7.24 ± 0.18	t = -0.397	0.692
Time course of stroke, years, Mean ± SE	8.94 ± 0.45	9.31 ± 0.50	8.03 ± 0.73	t = -1.556	0.123
Height, cm, Mean ± SE	165.37 ± 0.48	165.68 ± 0.62	164.61 ± 0.75	t = -1.056	0.294
Weight, kg, Mean ± SE	82.64 ± 1.19	84.47 ± 1.43	78.01 ± 1.62	t = -3.085	0.003
BMI, n (%)				χ^2^ = 2.865	0.094
Underweight/normal weight	198 (24.46)	115 (22.34)	83 (29.81)		
Overweight/obesity	667 (75.54)	472 (77.66)	195 (70.19)		
Waist circumference, n (%)				χ^2^ = 4.750	0.011
Low	244 (28.34)	162 (28.20)	82 (28.72)		
High	560 (65.14)	398 (67.49)	162 (59.21)		
Unknown	61 (6.52)	27 (4.32)	34 (12.07)		
SBP, mmHg, Mean ± SE	130.09 ± 1.10	129.08 ± 1.31	132.62 ± 2.08	t = 1.431	0.156
DBP, mmHg, Mean ± SE	68.62 ± 0.64	69.75 ± 0.75	65.72 ± 0.94	t = -3.465	0.001
TC, mg/dL, Mean ± SE	185.53 ± 2.35	182.90 ± 2.36	192.16 ± 4.16	t = 2.105	0.038
LDL-C, mg/dL, Mean ± SE	106.72 ± 2.48	105.64 ± 2.81	109.58 ± 4.20	t = 0.815	0.418
HDL-C, mg/dL, Mean ± SE	52.17 ± 0.95	52.22 ± 1.13	52.05 ± 1.63	t = -0.085	0.932
Non-HDL-C, mg/dL, Mean ± SE	133.36 ± 2.43	130.68 ± 2.29	140.10 ± 4.58	t = 2.022	0.046
TG, mg/dL, Mean ± SE	130.01 ± 7.20	126.30 ± 6.08	139.76 ± 18.76	t = 0.702	0.485
Fasting glucose, mg/dL, Mean ± SE	117.87 ± 3.08	119.25 ± 3.87	114.42 ± 4.58	t = -0.807	0.422
HbAlc, %, Mean ± SE	6.03 ± 0.05	5.97 ± 0.06	6.18 ± 0.08	t = 2.233	0.028
Cr, mg/dL, Mean ± SE	1.05 ± 0.02	0.98 ± 0.02	1.22 ± 0.04	t = 5.509	< 0.001
eGFR, mL/min/1.73m^2^, n (%)				χ^2^ = 55.306	< 0.001
≥ 6	660 (80.69)	500 (88.58)	160 (60.77)		
< 6	205 (19.31)	87 (11.42)	118 (39.23)		
HEI-2015, Mean ± SE	52.29 ± 0.56	51.44 ± 0.69	54.43 ± 1.06	t = 2.260	0.026
Health behaviors score, n (%)				χ^2^ = 1.283	0.278
< 43.75	276 (32.68)	194 (33.39)	82 (30.88)		
43.75–61.25	299 (30.17)	184 (27.76)	115 (36.25)		
≥ 61.25	290 (37.16)	209 (38.85)	81 (32.87)		
Health factors score, n (%)				χ^2^ = 0.841	0.433
< 47.50	284 (31.34)	192 (29.79)	92 (35.26)		
47.50–62.50	300 (31.46)	212 (31.36)	88 (31.71)		
≥ 62.50	281 (37.20)	183 (38.85)	98 (33.03)		
LE8 score, n (%)				χ^2^ = 2.319	0.104
< 48.123	301 (31.89)	206 (31.13)	95 (33.81)		
48.13–58.75	263 (28.05)	171 (26.19)	92 (32.75)		
≥ 58.75	301 (40.06)	210 (42.68)	91 (33.44)		
Survival status, n (%)				χ^2^ = 381.642	< 0.001
Survival	587 (71.62)	587 (100.00)	0 (0.00)		
Cardio-cerebrovascular mortality	89 (8.38)	0 (0.00)	89 (29.53)		
Others-cause mortality	189 (20.00)	0 (0.00)	189 (70.47)		

t: t test, χ^2^: chi-square test

*SE* Standard error, *PIR* Poverty income ratio, *DM* Diabetes mellitus, *CHF* Congestive heart failure, *CHD* Coronary heart disease, *BMI* Body mass index, *SBP* Systolic blood pressure, *DBP* Diastolic blood pressure, *TC* Total cholesterol, *LDL-C* Low-density-lipoprotein cholesterol, *HDL-C* High-density-lipoprotein cholesterol, *TG* Triglyceride, *HbAlc* Glycosylated hemoglobin, *Cr* Creatinine, *eGFR* Estimated glomerular filtration rate, *HEI-2015* The Healthy Eating Index 2015, *LE8* The Life's Essential 8

## Associations of LE8 with all-cause mortality and cardio-cerebrovascular mortality in patients with stroke

Table S2 showed characteristics respectively linked to all-cause mortality and cardio-cerebrovascular mortality. Age, race, cancer, CHF, CHD, anticoagulants, antiplatelet agents, waist circumference, and eGFR were significantly associated with all-cause mortality, whereas that age, race, sedentary time, cancer, CHF, CHD, anticoagulants, antiplatelet agents, and eGFR were associated with cardio-cerebrovascular mortality (all *P* < 0.05). Then, we constructed multivariate models on the basis of these variables, and screen the covariates through stepwise regression.

We investigated the association of LE8 with all-cause mortality in patients with stroke (Table 2). After adjusting for all covariates, including age, gender, race, education level, marital status, PIR, CHF, CHD and eGFR, patients with LE8 score ≥ 58.75 seemed to have lower risk of all-cause mortality compared to those with LE8 score < 48.13 (HR = 0.46, 95%CI: 0.31–0.69). Besides, health behaviors scores ≥ 61.25 (HR = 0.51, 95%CI: 0.32–0.81) and health factors scores of 47.50–62.50 (HR = 0.63, 95%CI: 0.40–1.00) were also associated with lower risk of all-cause mortality in patients with stroke.

**Table 2:** Association of LE8 with all-cause mortality in patients with stroke

Variables	Model 1		Model 2		Model 3	
	HR (95% CI)	*P*	HR (95% CI)	*P*	HR (95% CI)	*P*
LE8 score						
< 48.13	Ref		Ref		Ref	
48.13–58.75	1.00 (0.70–1.43)	0.999	0.87 (0.59–1.29)	0.485	1.01 (0.67–1.52)	0.964
≥ 58.75	0.65 (0.46–0.92)	0.015	0.43 (0.29–0.63)	< 0.001	0.46 (0.31–0.69)	< 0.001
Health behaviors score						
< 43.75	Ref		Ref		Ref	
43.75–61.25	1.38 (0.90–2.13)	0.140	0.70 (0.46–1.05)	0.082	0.82 (0.54–1.25)	0.360
≥ 61.25	1.01 (0.63–1.61)	0.970	0.47 (0.29–0.74)	0.001	0.51 (0.32–0.81)	0.004
Health factors score						
< 47.50	Ref		Ref		Ref	
47.50–62.50	0.78 (0.53–1.15)	0.216	0.62 (0.41–0.93)	0.019	0.63 (0.40–1.00)	0.048
≥ 62.50	0.64 (0.42–0.97)	0.036	0.61 (0.41–0.89)	0.011	0.69 (0.46–1.02)	0.066

Model 1: unadjusted model

Model 2: adjusted for age, gender, race, education level, marital status and PIR

Model 3: adjusted for age, gender, race, education level, marital status, PIR, CHF, CHD, and Egfr

*LE8* The Life's Essential 8, *HR* Hazard ratio, *CI* Confidence interval, *Ref* Reference

In addition, the Table 3 showed association of LE8 with cardio-cerebrovascular mortality in patients with stroke. Similarly, we found the highest tertile of LE8 score was linked to a lower risk of cardio-cerebrovascular mortality (HR = 0.51, 95%CI: 0.26–0.98) after adjusting for age, gender, race, education level, marital status, PIR, CHF, anticoagulants and eGFR. Also, health factors scores of 47.50–62.50 was associated with lower risk of cardio-cerebrovascular mortality (HR = 0.45, 95%CI: 0.22–0.92) compared to those < 47.50. Unfortunately, no significant association between health behaviors score and cardio-cerebrovascular mortality was observed (*P* > 0.05).

**Table 3:** Association of LE8 with cardio-cerebrovascular mortality in patients with stroke

Variables	Model 1		Model 2		Model 3	
	HR (95% CI)	*P*	HR (95% CI)	*P*	HR (95% CI)	*P*
LE8 score						
< 48.123	Ref		Ref		Ref	
48.13–58.75	0.86 (0.47–1.58)	0.637	0.78 (0.42–1.46)	0.434	0.98 (0.55–1.73)	0.937
≥ 58.75	0.61 (0.31–1.19)	0.149	0.40 (0.20–0.80)	0.009	0.51 (0.26–0.98)	0.043
Health behaviors score						
< 43.75	Ref		Ref		Ref	
43.75–61.25	1.37 (0.79–2.39)	0.263	0.73 (0.40–1.33)	0.310	0.91 (0.46–1.83)	0.801
≥ 61.25	1.19 (0.60–2.38)	0.613	0.61 (0.28–1.32)	0.208	0.69 (0.31–1.51)	0.349
Health factors score						
< 47.50	Ref		Ref		Ref	
47.50–62.50	0.48 (0.24–0.94)	0.032	0.39 (0.19–0.79)	0.009	0.45 (0.22–0.92)	0.028
≥ 62.50	0.47 (0.23–0.98)	0.044	0.43 (0.23–0.79)	0.007	0.58 (0.32–1.04)	0.068

Model 1: unadjusted model

Model 2: adjusted for age, gender, race, education level, marital status and PIR

Model 3: adjusted for age, gender, race, education level, marital status, PIR, CHF, anticoagulants and eGFR

*LE8* The Life's Essential 8, *HR* Hazard ratio, *CI* Confidence interval, *Ref* Reference

### Associations of LE8 with all-cause mortality and cardio-cerebrovascular mortality in subgroups

**Fig. 2:**
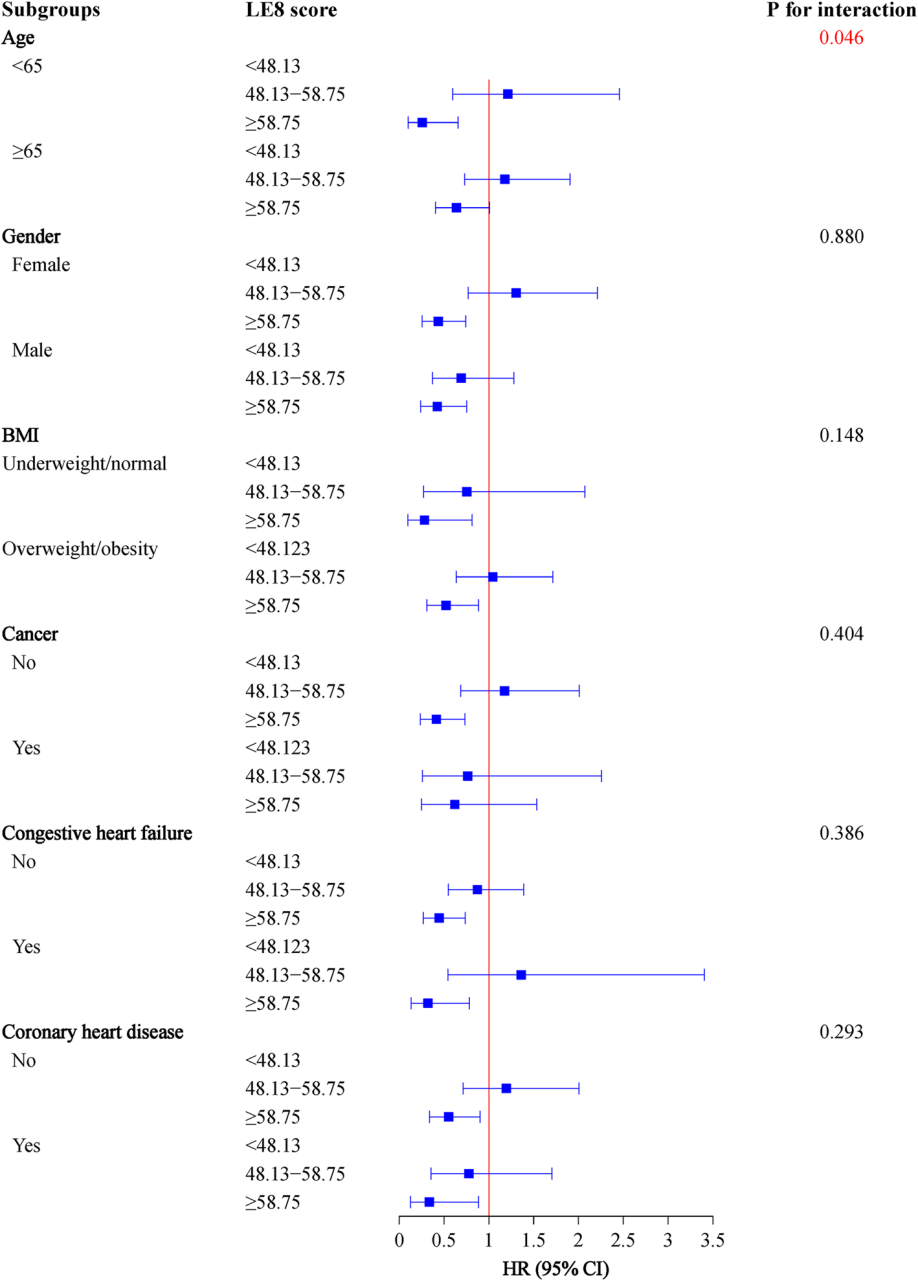
Association of LE8 with all-cause mortality in subgroups of age, gender, BMI, cancer, CHF, and CHD

**Fig. 3:**
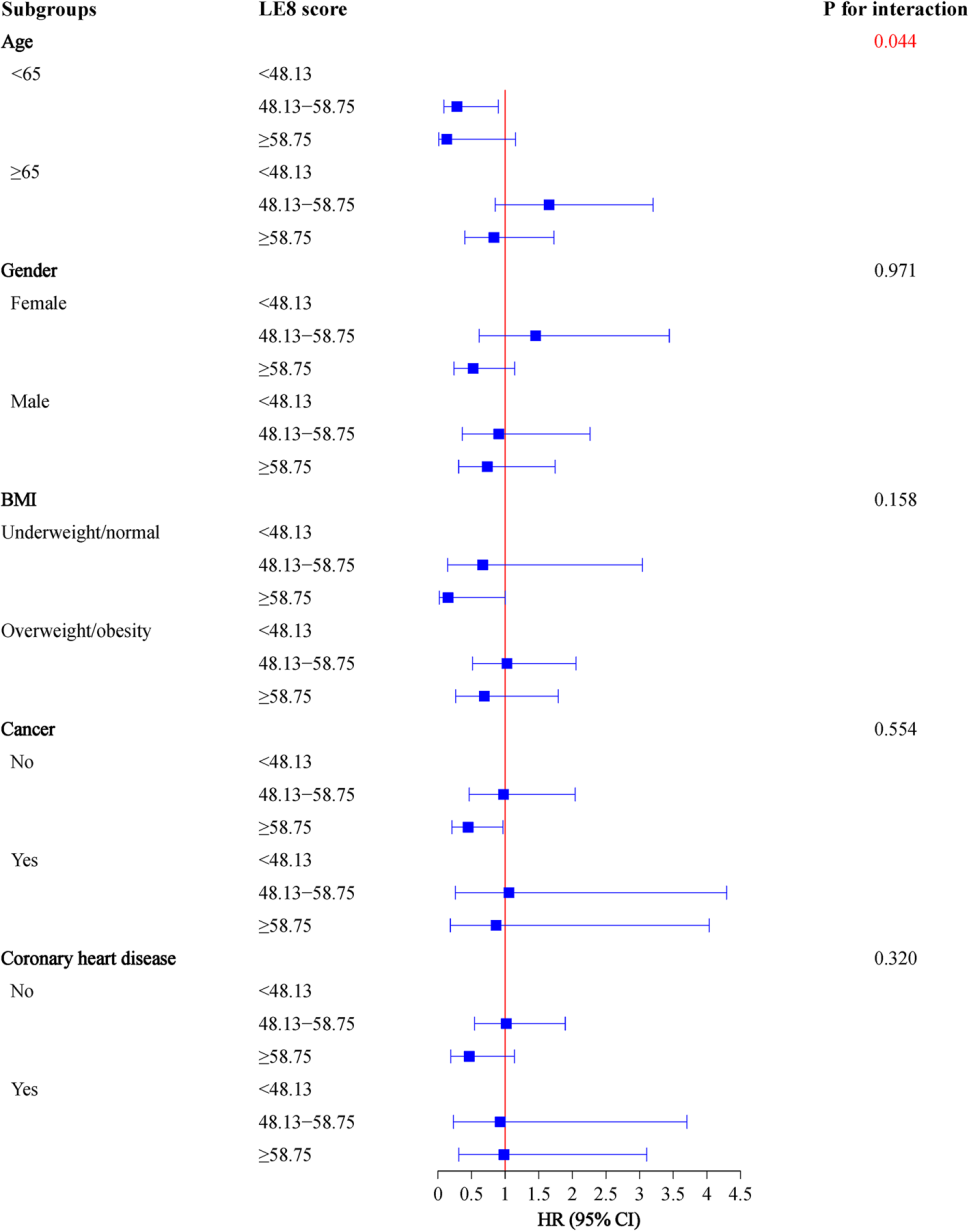
Association of LE8 with cardio-cerebrovascular mortality in subgroups of age, gender, BMI, cancer, and CHD

These associations were further explored in age, gender, BMI, cancer, CHF, and CHD subgroups. It’s easy to see in the Fig. 2, higher LE8 scores were associated with lower risk of all-cause mortality in patients aged < 65 years old (HR = 0.26, 95%CI: 0.10–0.66), without cancer (HR = 0.42, 95%CI: 0.24–0.73), and whatever the gender (female: HR = 0.44, 95%CI: 0.26–0.74; male: HR = 0.43, 95%CI: 0.24–0.75), BMI (underweight/normal: HR = 0.28, 95%CI: 0.10–0.81; overweight/obesity: HR = 0.52, 95%CI: 0.31–0.88), CHF (non-CHF: HR = 0.45, 95%CI: 0.27–0.74; CHF: HR = 0.32, 95%CI: 0.13–0.78) or CHD (non-CHD: HR = 0.55, 95%CI: 0.34–0.90; CHD: HR = 0.34, 95%CI: 0.13–0.88) conditions. Differently, the negative relationship between LE8 scores and cardio-cerebrovascular mortality were found in age < 65 years old (HR = 0.28, 95%CI: 0.09–0.90) and non-cancer (HR = 0.45, 95%CI: 0.21–0.97) subgroups (Fig. 3).

## Discussion

In the current study, we investigated the associations of LE8 with all-cause mortality and cardio-cerebrovascular mortality in patients with stroke respectively. The study results suggested that a higher LE8 score was linked to both lower risk of all-cause mortality and cardio-cerebrovascular mortality. The negative association between LE8 and all-cause mortality was found in patients aged < 65 years old, without cancer, and whatever the gender, BMI, CHF or CHD conditions. In addition, the relationship between a higher LE8 score and low cardio-cerebrovascular mortality risk was only observed in aged < 65 years old and non-cancer subgroups.

The LE8 metric was introduced on the basis of LS7, adding sleep metrics and revisions of the previous 7 domains, and has been reported to be associated with incident CCVDs as well as mortality [9, 19]. Due to the severe disease burden of stroke globally, we thought it is necessary to clarify the association of LE8 with mortality risk in patients with stroke in order to provide some references for relatively comprehensive methods on stroke prevention and management. Studies have investigated the role of LE8 in all-cause and cardio-cerebrovascular mortality in different populations. Hernández-Martínez et al. [20] assessed the association of LE8 with mortality in the Spanish adult population, and found that a higher LE8 score was associated with lower all-cause and CVD related mortality. Isiozor et al. [21] examined the association between LE8 and the risk of cardiovascular and all-cause mortality in Finnish men, and showed that high level of CVH, which was defined by LE8, was linked to significantly lower risks of CHD, stroke, as well as CVD. Different to these previous studies, our study population was adult patients with stroke in the United States, which relatively supplemented the literature blank in this population.

The LE8-related CVH score can significantly predict future stroke risk [11]. Smoking, non-HDL-C, blood pressure, BMI, HbA1c, physical activity, diet quality, and sleep duration were used to create a modified version of the LE8 score. Among the above 8 factors, we found physical activity, BMI, and blood pressure had the highest contribution to the predictive value of LE8 on all-cause mortality in patients with stroke (Figure S1), whereas physical activity, sleep duration, and diet quality had the highest contribution to that on cardio-cerebrovascular mortality (Figure S2). A prospective cohort study has shown that objectively measured physical activity was linked to lower risk of stroke or all-cause mortality in 70-year-old individuals [22]. Also, physical activity was strongly associated with lower risk of CVD-, CHD-, and stroke-related mortality among people with a history of these specific diseases [23]. Therefore, advocating appropriate physical activity in stroke patients is an effective measure to reduce the risk of poor prognosis. In adults, a higher BMI is linked with a higher chance of hypertension, CVD, stroke, heart disease, and mortality [24–26]. It seemed that the predictive value of cardiovascular-related factors for all-cause mortality in stroke patients was relatively higher, but the specific mechanisms need further clarification. In addition to physical activity, sleep duration and diet quality also played important roles in the association between IE8 and cardio-cerebrovascular mortality in stroke patients. In fact, sleep was a new metric added into LE8 [7]. A prospective magnetic resonance neuroimaging study in middle-aged individuals without stroke or dementia enrolled in the UK Biobank showed that suboptimal sleep duration is associated with poorer neuroimaging brain health profiles in middle-aged adults without stroke or dementia [27]. Similarly, a high diet quality, especially adherence to the Mediterranean (Med) diet pattern, can effectively reduce cardiovascular mortality [28]. Livingstone et al. [29] considered a better quality of diet predicted lower risk of all-cause and CVD related mortality in Australian adults, while a more inflammatory diet predicted higher mortality risk.

The LE8 provides a simple and effective tool for assessing individual’s CVH and promoting healthy lifestyle behaviors. The LE8 metrics would not only benefit individuals to specifically identify modifiable risk factors but would highlight LE8 factors that can be directly changed for better and improved LE8 scores and better CVH [30]. However, the underlying mechanisms that a higher LE8 linked to a better prognosis of stroke are still unclear. Previous studies have concluded several hypothetical mechanisms. Hypertension may be indirectly related to white matter hyperintensities (WMH) progression via arterial compliance, and the underlying pathophysiological process between hypertension and mortality in stroke may be associated with cognitive impairment associated with stroke [31]. In parallel, hyperlipidaemia results in microvascular haemodynamic regulation disorder, and increases viscosity and resistance of blood flow, for the progression of WMH [32]. Besides, visceral obesity contributed to deep WMH through increases in proinflammatory cytokines [33]. The healthy dietary patterns can improve endothelial function, adiposity and lower levels of inflammatory markers [34]. Meanwhile, poor sleep efficiency was independently associated with basal ganglia according to altering waste clearance mechanisms, and sleep may be associated with relieving inflammation [35]. Healthcare providers could use LE8 to educate stroke patients on the importance of maintaining a healthy lifestyle and managing risk factors such as blood pressure, physical activity levels, and sleep duration. However, as the current study did not find significant associations between health behaviors and cardio-cerebrovascular mortality, indicating that stroke patients are recommend to focus on improving the overall LE8 score rather than emphasizing only one component.

Furthermore, subgroup analyses showed associations of LE8 with all-cause mortality and cardio-cerebrovascular mortality had differences in gender and cancer subgroups. In general population in the United States, no gender difference has been observed according to previous researches on association between LE8 and mortality risk [8, 36]. In 2019, stroke was the 3rd leading cause of mortality in female adults, compared with 5th in males in the United States [37]. Also, elevated BMI, clinical obesity, and higher waist circumference are linked to higher risk of total and ischemic stroke in both sexes, with a stronger association in women than men [38]. Female-specific risk factors such as use of oral contraceptive pills, menopausal hormone therapy, and adverse pregnancy outcomes, may also increase the possibility of adverse prognoses in stroke [39]. Therefore, our findings indicated that targeted health promotion strategies and lifestyle changes should be used in women with stroke to reduce the risk of mortality. Additionally, compared with patients with cancer, higher LE8 scores in those who without cancer were significantly associated with lower risk of both all-cause mortality and cardio-cerebrovascular mortality. It may because these populations may be particularly concerned about intervening with lifestyle-related factors or following medical advice for specific treatments in clinical practice.

This was the first research investigated the associations of LE8 with all-cause mortality and cardio-cerebrovascular mortality in patients with stroke on the basis of the NHANES database, which includes large samples with good representativeness. Our results may provide some references for further comprehensive health management in stroke survivors to reduce the disease burdens. Nevertheless, there are some limitations in this study. Although we have taken possible confounding factors into consideration, due to some of the data were self-reported, and unavailable follow-up duration data, information bias is unavoidable. In addition, the study populations were only from the United States that the applicability of LE8 in stroke populations of other races still needs to be further validated.

## Conclusion

A high LE8 score was associated with lower risk of mortality in stroke patients, which may provide some information on prognosis management in stroke. However, the causal association between LE8 and stroke prognosis needs further clarification.

### Supplementary Information

Supplementary Material 1. 

Supplementary Material 2: Figure S1. Contributions of each component in the association between LE8 and all-cause mortality.

Supplementary Material 3: Figure S2. Contributions of each component in the association between LE8 and cardio-cerebrovascular mortality. 

## Data Availability

The datasets generated during and/or analyzed during the current study are available in the NHANES database, https://ftp.cdc.gov/pub/health_statistics/NCHS/datalinkage/linked_mortality.

## Abbreviations

CCVDs: Cardio-cerebrovascular diseases
DM: Diabetes mellitus
AHA: American Heart Association
LS7: Life’s Simple 7
CVH: Cardiovascular health
LE8: Life’s Essential 8
BMI: Body mass index
NHANES: National Health and Nutrition Examination Surveys
NCHS: National Center for Health Statistics
CDC: Centers for Disease Control and Prevention
MECs: Mobile exam centers
CHF: Congestive heart failure
CHD: Coronary heart disease
IRB: Institutional review board
PIR: Poverty income ratio
SBP: Systolic blood pressure
DBP: Diastolic blood pressure
TC: Total cholesterol
LDL-C: Low-density-lipoprotein cholesterol
TG: Triglyceride
HbAlc: Glycosylated hemoglobin
Cr: Creatinine
eGFR: Estimated glomerular filtration rate
HEI: Healthy Eating Index
USDA: United States Department of Agriculture
WHO: World Health Organization
NDI: National Death Index
HRs: Hazard ratios
CIs: Confidence intervals
