# Supplementary material for: Association of Life’s essential 8 score with the risk of all-cause mortality and cardio-cerebrovascular mortality in patients with stroke
Source: BMC Cardiovasc Disord. 2024 Jun 25;24:320. doi: 10.1186/s12872-024-03947-3 (PMC11197366; doi:10.1186/s12872-024-03947-3)
Supplement: Supplementary file 1 — Supplementary Material 1. [file 12872_2024_3947_MOESM1_ESM.docx]

**Table S1.** **Score of each component in LE8 between survival group and all-cause mortality group**

| Variables | Total  (N=865) | Survival (N=587) | All-cause mortality (N=278) | Statistics | *P* |
| --- | --- | --- | --- | --- | --- |
| **Health behaviors** |  |  |  |  |  |
| Diet quality, Mean ± SE | 44.18 ± 1.28 | 42.20 ± 1.58 | 49.17 ± 2.42 | t = 2.311 | 0.023 |
| Physical activity, Mean ± SE | 32.91 ± 2.68 | 38.41 ± 3.49 | 19.04 ± 3.12 | t = -3.953 | <0.001 |
| Tobacco exposure, Mean ± SE | 60.09 ± 1.89 | 57.98 ± 2.16 | 65.40 ± 3.70 | t = 1.715 | 0.090 |
| Sleep duration, Mean ± SE | 72.04 ± 1.59 | 71.80 ± 1.82 | 72.65 ± 3.38 | t = 0.219 | 0.827 |
| **Health factors** |  |  |  |  |  |
| BMI, Mean ± SE | 54.21 ± 1.96 | 51.62 ± 2.30 | 60.73 ± 2.66 | t = 2.844 | 0.006 |
| Non-HDL-C, Mean ± SE | 62.43 ± 1.69 | 64.53 ± 1.64 | 57.13 ± 3.08 | t = -2.306 | 0.023 |
| Blood glucose, Mean ± SE | 64.29 ± 1.23 | 65.98 ± 1.58 | 60.00 ± 1.82 | t = -2.407 | 0.018 |
| Blood pressure, Mean ± SE | 48.75 ± 1.82 | 51.56 ± 2.27 | 41.66 ± 2.96 | t = -2.640 | 0.010 |

LE8: the Life's Essential 8, SE: standard error, BMI: body mass index, HDL-C: high-density-lipoprotein cholesterol.

**Table S2. Characteristics associated with all-cause mortality and cardio-cerebrovascular mortality respectively**

| Variables | All-cause mortality | | Cardio-cerebrovascular mortality | |
| --- | --- | --- | --- | --- |
|  | HR (95% CI) | *P* | HR (95% CI) | *P* |
| Age | 1.08 (1.06-1.10) | <0.001 | 1.08 (1.04-1.12) | <0.001 |
| Gender |  |  |  |  |
| Female | Ref |  | Ref |  |
| Male | 1.03 (0.75-1.41) | 0.869 | 1.33 (0.80-2.20) | 0.278 |
| Race |  |  |  |  |
| Non-Hispanic white | Ref |  | Ref |  |
| Non-Hispanic black | 0.69 (0.47-1.01) | 0.057 | 0.94 (0.55-1.61) | 0.826 |
| Mexican American | 0.62 (0.28-1.35) | 0.227 | 0.80 (0.28-2.26) | 0.670 |
| Others | 0.40 (0.17-0.93) | 0.032 | 0.09 (0.02-0.45) | 0.004 |
| Education level |  |  |  |  |
| Less than high school | Ref |  | Ref |  |
| High school | 0.91 (0.61-1.35) | 0.642 | 0.78 (0.40-1.52) | 0.466 |
| Above high school | 0.71 (0.47-1.06) | 0.093 | 0.80 (0.42-1.53) | 0.500 |
| Marital status |  |  |  |  |
| Married/living with partner | Ref |  | Ref |  |
| Never married/divorced/separated/widowed | 1.32 (0.89-1.95) | 0.166 | 1.29 (0.71-2.34) | 0.407 |
| PIR |  |  |  |  |
| ≤1.3 | Ref |  | Ref |  |
| >1.3 | 1.27 (0.85-1.89) | 0.237 | 1.58 (0.85-2.96) | 0.152 |
| Unknown | 1.86 (0.95-3.65) | 0.072 | 1.69 (0.40-7.20) | 0.476 |
| Heavy alcohol drinking |  |  |  |  |
| No | Ref |  | Ref |  |
| Yes | 0.72 (0.37-1.42) | 0.345 | 0.69 (0.23-2.10) | 0.514 |
| Unknown | 0.91 (0.61-1.34) | 0.623 | 0.93 (0.52-1.65) | 0.801 |
| Sedentary time |  |  |  |  |
| <4 | Ref |  | Ref |  |
| ≥4 | 1.52 (0.94-2.44) | 0.084 | 2.88 (1.28-6.45) | 0.010 |
| Depression |  |  |  |  |
| No | Ref |  | Ref |  |
| Yes | 0.76 (0.55-1.03) | 0.080 | 0.85 (0.48-1.49) | 0.566 |
| Cancer |  |  |  |  |
| No | Ref |  | Ref |  |
| Yes | 2.40 (1.65-3.48) | <0.001 | 2.68 (1.46-4.93) | 0.001 |
| CHF |  |  |  |  |
| No | Ref |  | Ref |  |
| Yes | 1.75 (1.10-2.78) | 0.018 | 2.87 (1.70-4.83) | <0.001 |
| CHD |  |  |  |  |
| No | Ref |  | Ref |  |
| Yes | 1.82 (1.12-2.96) | 0.016 | 2.69 (1.32-5.51) | 0.007 |
| Anticoagulants |  |  |  |  |
| No | Ref |  | Ref |  |
| Yes | 2.20 (1.44-3.37) | <0.001 | 4.58 (2.83-7.43) | <0.001 |
| Antiplatelet agents |  |  |  |  |
| No | Ref |  | Ref |  |
| Yes | 1.47 (1.04-2.07) | 0.029 | 1.83 (1.10-3.04) | 0.020 |
| Time course of stroke | 0.99 (0.97-1.01) | 0.353 | 1.00 (0.98-1.03) | 0.748 |
| Waist circumference |  |  |  |  |
| Low | Ref |  | Ref |  |
| High | 1.09 (0.70-1.70) | 0.688 | 1.11 (0.56-2.18) | 0.773 |
| Unknown | 3.75 (1.96-7.18) | <0.001 | 1.74 (0.62-4.90) | 0.294 |
| eGFR |  |  |  |  |
| ≥6 | Ref |  | Ref |  |
| <6 | 3.91 (2.96-5.18) | <0.001 | 3.98 (2.21-7.18) | <0.001 |

HR: hazard ratio, CI: confidence interval, Ref: reference, PIR: poverty income ratio, CHF: congestive heart failure, CHD: coronary heart disease, eGFR: estimated glomerular filtration rate.
