# Supplementary figures and images for: Association of Life’s essential 8 score with the risk of all-cause mortality and cardio-cerebrovascular mortality in patients with stroke
Source: BMC Cardiovasc Disord. 2024 Jun 25;24:320. doi: 10.1186/s12872-024-03947-3 (PMC11197366; doi:10.1186/s12872-024-03947-3)

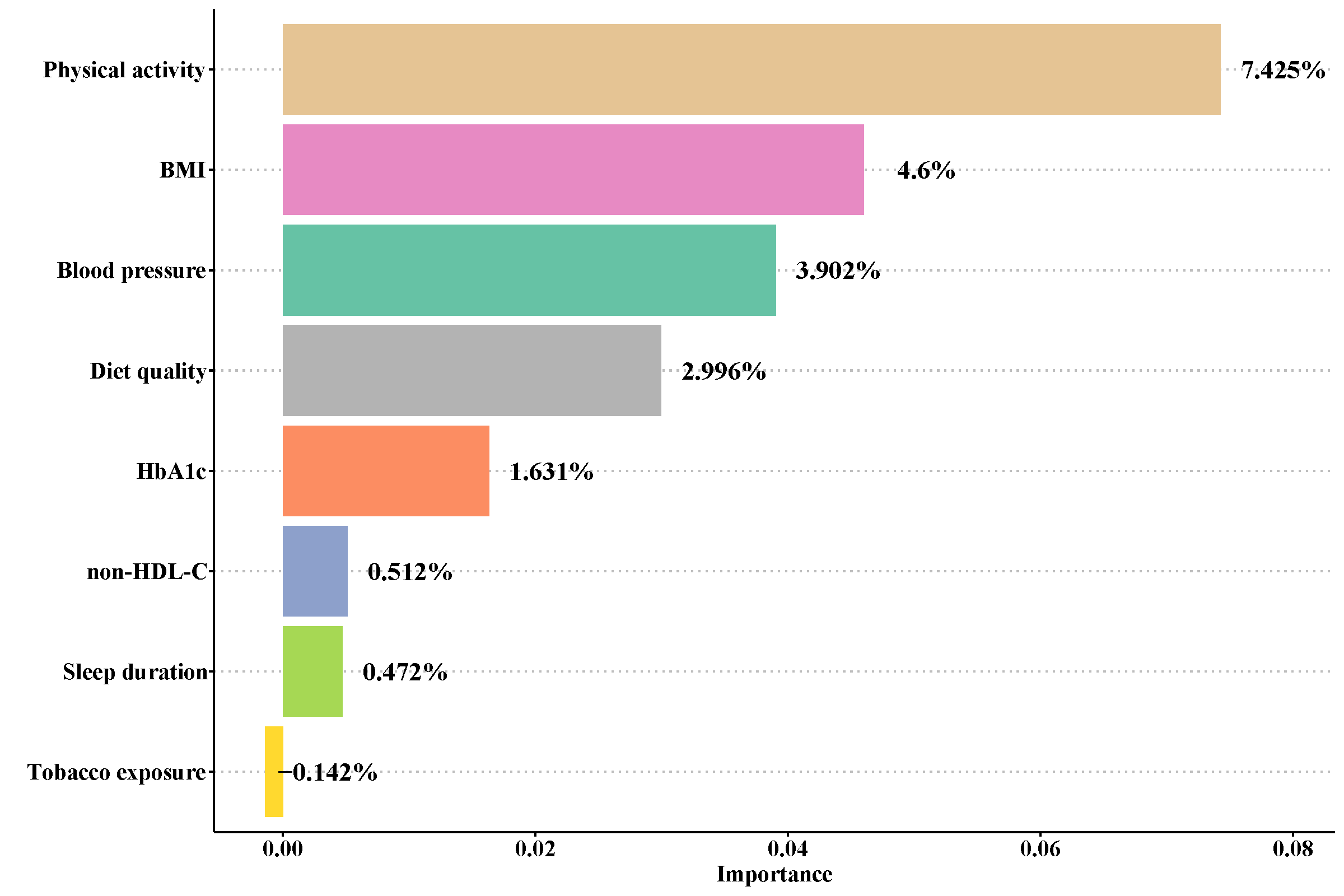

Supplement: Supplementary file 2 — Supplementary Material 2: Figure S1. Contributions of each component in the association between LE8 and all-cause mortality. [file 12872_2024_3947_MOESM2_ESM.tiff]

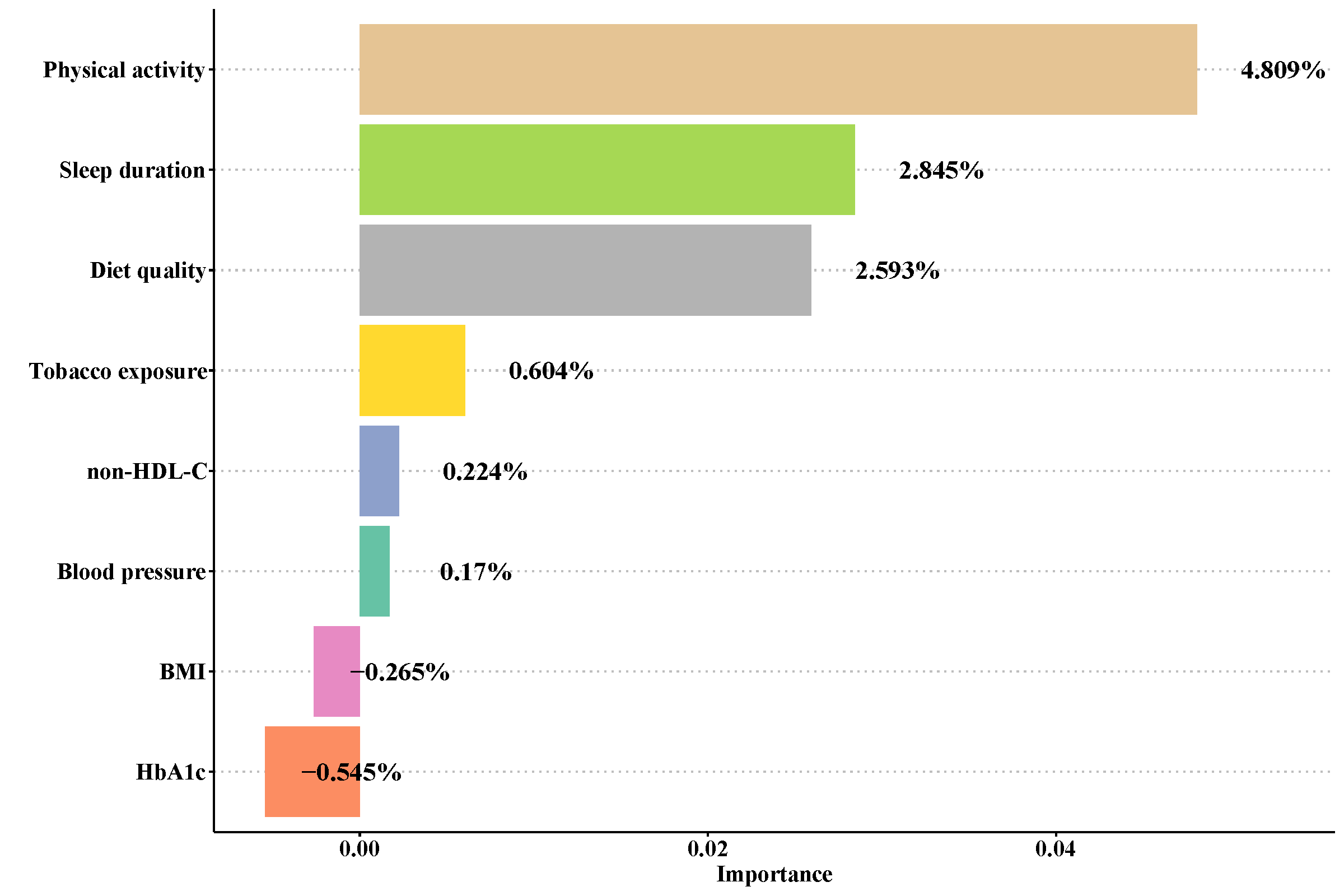

Supplement: Supplementary file 3 — Supplementary Material 3: Figure S2. Contributions of each component in the association between LE8 and cardio-cerebrovascular mortality. [file 12872_2024_3947_MOESM3_ESM.tiff]
